# Exploring the Mechanisms of LiNiO_2_ Cathode Degradation by the Electrolyte Interfacial Deprotonation Reaction

**DOI:** 10.1021/acsami.4c10458

**Published:** 2024-10-04

**Authors:** Yu Zheng, Perla B. Balbuena

**Affiliations:** ^†^Department of Chemical Engineering, ^‡^Department of Chemistry, ^§^Department of Materials Science and Engineering, Texas A&M University, College Station, Texas 77843, United States

**Keywords:** LiNiO_2_, Ni dissolution, electrolyte deprotonation, cathode degradation, ab initio molecular dynamics, lithium-ion batteries

## Abstract

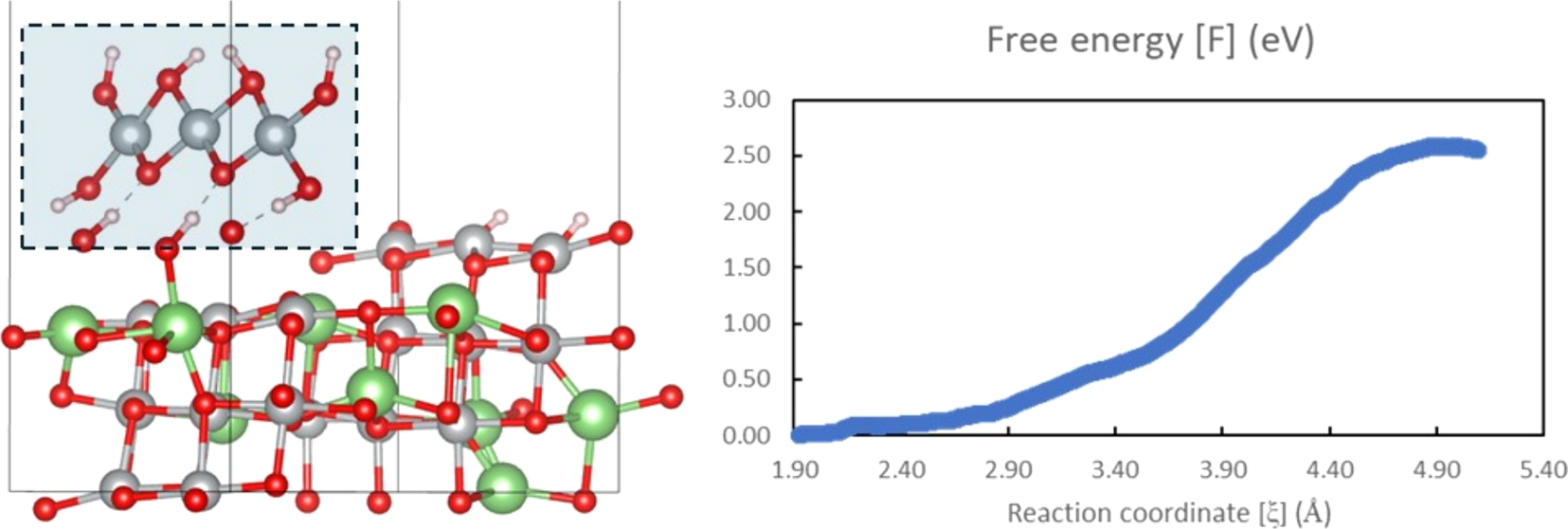

Nickel-rich layered oxides stand as ideal cathode candidates for high specific capacity and energy density next-generation lithium-ion batteries. However, increasing the Ni content significantly exacerbates structural degradation under high operating voltage, which greatly restricts large-scale commercialization. While strategies are being developed to improve cathode material stability, little is known about the effects of electrolyte–electrode interaction on the structural changes of cathode materials. Here, using LiNiO_2_ in contact with electrolytes with different proton-generating levels as model systems, we present a holistic picture of proton-induced structural degradation of LiNiO_2_. Through ab initio molecular dynamics calculations based on density functional theory, we investigated the mechanisms of electrolyte deprotonation, protonation-induced Ni dissolution, and cathode degradation and the impacts of dissolved Ni on the Li metal anode surfaces. We show that the proton-transfer reaction from electrolytes to cathode surfaces leads to dissolution of Ni cations in the form of NiOOH_*x*_, which stimulates cation mixing and oxygen loss in the lattice accelerating its layered-spinel–rock-salt phase transition. Migration of dissolved Ni^2+^ ions to the anode side causes their reduction into the metallic state and surface deposition. This work reveals that interactions between the electrolyte and cathode that result in protonation can be a dominant factor for the structural stability of Ni-rich cathodes. Considering this factor in electrolyte design should be of benefit for the development of future batteries.

## 1 Introduction

Layered Ni-rich oxides such as LiNiO_2_ are among the most promising cathode materials for lithium-ion batteries (LIBs) because of their high capacity. However, Ni-rich cathodes inherently exhibit severe instability (chemical, electrochemical, structural, and thermal) issues, causing irreversible capacity fading of LIBs, which restricts their large-scale commercialization. It was confirmed that Ni-rich cathode materials are prone to irreversible transformation during the delithiation process, resulting in rapid performance fading.^1^ The degree of degradation depends on the elemental composition of the cathode material. However, all Ni-rich cathodes undergo similar electronic and structural changes. This includes cation disorder, electrolyte decomposition, O_2_ and CO_2_ gas release, phase transition, and dissolution of transition metals (TMs).^2^ Cation disorder can alter the average voltage of Ni-rich layered cathodes,^3^ and electrolyte decomposition can cause electrode swelling.^4^ Gas release is a severe risk of catastrophic self-combustion of the battery.^5^ Phase transition results in blocking Li-ion diffusion paths and reducing ionic conductivity.^2^ Dissolved TMs can migrate through the electrolyte phase to the anode side causing “cross-talk” effects.^6^

Despite the clear correlation between cathode degradation and capacity fading of the battery, the effect of electrolyte–electrode interaction on cathode structure breakdown is still insufficient, not to mention the study of underlying molecular processes. Previous studies were carried out to investigate the effect of protonation on the structural changes of Ni-rich sodium cathodes^7^ and provided important atomistic insights into proton-induced phase transition from layered to rock salt. It was demonstrated that Ni-rich cathodes exhibit significantly different degradation rates in a localized high-concentration electrolyte (LHCE) and a conventional carbonate electrolyte.^8−10^ However, other consequences, such as Ni dissolution mechanisms and their consequences on cathode degradation and cross-talk events, were not studied.

In this work, we aim to reveal the molecular process of electrolyte decomposition on LiNiO_2_ cathode surfaces and proton-induced structural changes. We used a carbonate-based electrolyte and a LHCE as model electrolyte systems to investigate electrolyte–electrode interactions. Ab initio molecular dynamics (AIMD) simulations were performed to elucidate the cathode structure degradation associated with electrolyte deprotonation. The mechanisms of Ni dissolution were studied using constrained AIMD (c-AIMD) to investigate the details of surface reconstruction after metal dissolution and determine the role of the protonated surface atoms in the process, which may lead to establishing a relationship between the electrode/electrolyte chemistry and cathode stability. The results provide new insights into the correlation between cathode fading and electrolyte–electrode interactions and opens up new dimensions in engineering the cathode and electrolyte for future battery improvement.

## 2 Computational Methods

### 2.1 First-Principles Calculations

The first-principles calculations were performed using the density functional theory (DFT) method as implemented in the Vienna *ab initio* simulation package (VASP) code.^11−13^ The projector-augmented wave (PAW)^14,15^ method was used for the pseudopotentials of all atoms. The wave functions for valence electrons were expanded in a plane-wave basis set with 520 eV as the kinetic energy cutoff. To account for the localized nature of Ni 3d electrons, the DFT + *U* method was used.^16^ The *U* values were taken as 6.2 eV for Ni atoms.^17^ The generalized gradient approximation with the Perdew–Burke–Ernzerhof (GGA-PBE) functional^14,15,18^ was applied to evaluate the exchange-correlation energy. Van der Waals interactions were considered using the DFT–D3 correction method proposed by Grimme et al.^19,20^ The convergence criteria in the structure relaxations for the electronic self-consistent iterations and the ionic relaxation loops were set to be 10^–5^ eV and 0.02 eV/Å, respectively.

### 2.2 LiNiO_2_ Cathode Surface Model

The LiNiO_2_ (1 × 2 × 3) supercell was constructed with the *R*3̅*m* space group corresponding to 24 replications of the LiNiO_2_ formula unit. The (104) facet of LiNiO_2_ was chosen to build the surface slabs due to its lowest surface energy.^21^ On the basis of previous experimental and computational studies, (001) and (104) surfaces account for most of the exposed surface of LiNiO_2_ cathode particles.^22^ Here, we limit our analysis to (104) surfaces because the (104) facet intersects with the Li layers in the LiNiO_2_ crystal structure and thus permits Li^+^ intercalation/deintercalation. As discussed later, cathode degradation is facilitated at delithiated states; therefore, this is a convenient facet for our study. The surfaces were modeled with a thickness of 4 layers and separated by a vacuum gap of 15 Å along the direction perpendicular to the surface to eliminate spurious interactions across the periodic boundary condition. The bottom two layers were fixed to simulate the bulk cathode. A conjugate-gradient algorithm was employed to relax the ions into their instantaneous ground state. The Bader decomposition method was used for charge analysis.^23−25^

### 2.3 Electrolyte Decomposition on LiNiO_2_ Surfaces

Simulations of electrolyte decomposition on LiNiO_2_ surfaces were carried out following our previous study.^7^ A set of 10 ps AIMD simulations each with a constant number of electrons removed (*n*_re_ = 1, 2, 3, 4, 5, 7, 9, and 11), as presented in Figure S1(b), were performed to observe bulk electrolyte oxidative decomposition mechanisms. Since all electrolyte molecules were identified to participate reactions with *n*_re_ = 11 in the bulk electrolyte simulations, electron-deficient environments on surfaces were created by removing 12 electrons from the simulation cell as seen in Figure S1(c). 10 ps AIMD simulations were carried out to investigate LHCE [1 M LiFSI/DME/BTFE (1:1:3 molecular number ratio)] and EC/PC [1 M LiPF_6_/EC/PC (EC/PC 1:1 vol %)] on LiNiO_2_ (104) surfaces. The geometry optimization of individual electrolyte components was performed by Gaussian16 (G16) simulation software^12^ with the B3LYP hybrid functional and 6-311+G(d,p) basis set. The solvation effects were represented by the polarizable continuum model (PCM) with tetrahydrofuran (THF) as the solvent. The energetic calculations of electrolyte decomposition reactions were performed at the same level of theory.

### 2.4 Ni Dissolution from LiNiO_2_ Surfaces

Dissolution modeling was performed within the Born–Oppenheimer ab initio molecular dynamics methodology. The systems under study were initially equilibrated for 10 ps using a time step of 1 fs. The Nose–Hoover thermostat was used to keep the simulation temperature around 300 K. The slow-growth approach^26^ with a velocity of 0.5 Å/ps was employed to explore the Ni dissolution pathway. The bond distance between the dissolving Ni and surface O atoms was chosen as the reaction coordinate. The blue moon ensemble approach^27^ was employed to explore the free-energy landscape of the dissolution process. Each slow-growth trajectory was split into a set of windows with a spacing of ∼0.1 Å, and the atomic configuration in each window was then equilibrated for 2 ps. Each equilibration was followed by 2 ps of production run to collect force gradients along the constrained reaction coordinate. Finally, the free-energy profile was calculated through thermodynamic integration of the average force gradients along the reaction path.

### 2.5 Reduction of Dissolved Ni^2+^ on LMA Surfaces

1 M LHCE with one NiF_3_^–^ fragment was put in contact with the (100) facet of a Li metal slab to simulate the evolution of dissolved Ni^2+^ on lithium-metal anode (LMA) surfaces. A similar approach has been employed in the study of LHCE decomposition on LMA surfaces.^28^ 10 ps AIMD simulations were run at 330 K with a 1 fs time step within the NVT ensemble. A 9-layer (3 × 3) supercell model with the middle three layers fixed was employed to represent the LMA surface.

## 3 Results and Discussion

### 3.1 Electrolyte Deprotonation on LiNiO_2_ Surfaces

**Figure 1:**
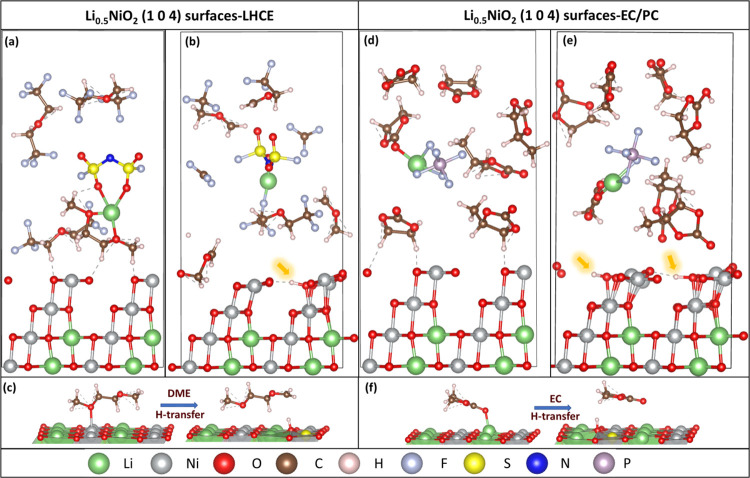
Electrolyte interfacial reactions on Li_0.5_NiO_2_ (104) surfaces. (a–c) LHCE decomposition: (a) initial configuration; (b) final configuration after 10 ps of AIMD simulations; (c) mechanism of DME proton-transfer reaction. (d–f) EC/PC decomposition: (d) initial configuration; (e) final configuration after 10 ps of AIMD simulations; (f) mechanism of EC proton-transfer reaction. Color code: lithium, green; nickel, gray; oxygen, red; carbon, brown; hydrogen, light pink; fluorine, light blue; sulfur, yellow; nitrogen, blue; phosphorus, light purple. Orange arrows in the simulation cell of final configurations (b, e) highlight the locations of surface protonation.

We first examined the electrolyte decomposition reactivities on the LiNiO_2_ (104) surfaces upon charging, following the simulation scheme shown in Figure S1. To model the effect of an electrified cathode interface, a number of electrons were removed from the simulation cell to mimic the electron-deficient environment near the cathode surface, as depicted in Figure S1(a).^29^ Previously, we used the same method to explore electrolyte decomposition mechanisms on the NaNiO_2_ cathode.^7^ Two electrolytes, LHCE and EC/PC, were studied to explore the effects of the solvation structure on the electrolyte stability. EC/PC is a conventional electrolyte where Li^+^ ions are solvated by the solvent molecules forming solvent-separated ion pairs (SSIPs), whereas LHCE is a diluted high-concentration electrolyte in which Li^+^ ions and FSI^–^ anions are jointly coordinated to solvent molecules, forming contact ion pairs (CIPs), whereas diluents are usually found in the second shell. When close to the surface, EC and PC can easily interact with the surface and deliver protons to the exposed atoms (Figure 1d,e). In contrast, in LHCE, even when the solvent can occasionally separate from the Li ion and protonate the surface (Figure 1a,b), the frequency is lower. This is consistent with the energetics of the deprotonation reaction, where EC/PC is more reactive than LHCE as shown in Figure S2.

Figure 1 demonstrates the initial and final configurations of LHCE and EC/PC decomposition on delithiated Li_0.5_NiO_2_ (104) surfaces. The simulation detected proton-transfer reactions from electrolyte molecules to the surface O (O_s_) atoms, leading to the formation of hydroxyl (−OH) groups on the surfaces, as highlighted with orange arrows in Figure 1b,e. The deprotonation of DME is shown in Figure 1c with the donation of the terminal hydrogen atom to the O_s_ atom. EC and PC transfer ethylene protons to the surfaces, as shown in Figure 1e,f. Similar carbonate deprotonation reactions were also reported on other transition-metal oxides and were suggested to lead to the capacity loss of LIBs.^30^

To compare the reactivities of LHCE and EC/PC deprotonation, we calculated the energetics of electrolyte deprotonation on both pristine and delithiated LiNiO_2_ (104) surfaces. As shown in Figure S2, electrolyte deprotonation is facilitated by Li^+^ dissolution (surface delithiation upon charging) because the reactions were found to be thermodynamically unfavorable on Li_1.0_NiO_2_ (104) surfaces and became favorable when the surfaces were partially delithiated. The reaction energies were calculated to be more negative following the order: PC > EC > DME > BTFE, which suggests the lower reactivity of LHCE deprotonation compared to EC/PC.

We next analyzed the change of the oxidation states of Ni atoms upon surface protonation by using DME deprotonation on Li_1.0_NiO_2_ and Li_0.5_NiO_2_ (104) surfaces as an example. Table S1 gives the magnetic moments and the corresponding oxidation states of Ni atoms upon proton-transfer reactions. It was revealed that a Ni atom (highlighted in yellow in Figure S3(a)) was reduced from the oxidation state of +3 to +2 when a proton was transferred to its neighboring O_s_ atom on the Li_1.0_NiO_2_ (104) surface, which is in accordance with the experimental observation of electron energy loss spectroscopy (EELS) of LiNiO_2_.^31^ As protonation continued during cathode delithiation, the reduction of Ni atoms also remained on the Li_0.5_NiO_2_ (104) surface, while some of the Ni ions increased to high oxidation states, from Ni^3+^ to Ni^4+^, as shown in Figure S3(b). Meanwhile, the Ni–O bond length increased from 1.946 to 2.132 Å upon surface protonation, which represents the weakening of the Ni–O bonding strength.

### 3.2 Mechanisms of Ni Dissolution from Protonated LiNiO_2_ Surfaces

It is recognized that the cathode surface becomes more oxidative upon delithiation, promoting protonation of surface oxygen atoms, weakening TM–O bonds, and facilitating TM dissolution.^32^ However, the mechanisms of Ni dissolution are not completely understood. To unveil the dissolution mechanism, we employed c-AIMD simulations to evaluate the free-energy landscape of surface Ni atoms migrating from the surface-bound state to the dissolved state. The bond length between the surface Ni atom and the subsurface O atom (O_sub_) was chosen as the reaction coordinate. The simulations were performed while constraining the Ni–O_sub_ bond distance following a previous study of Mn dissolution from Li_*x*_Mn_2_O_4_ surfaces.^33^

**Figure 2:**
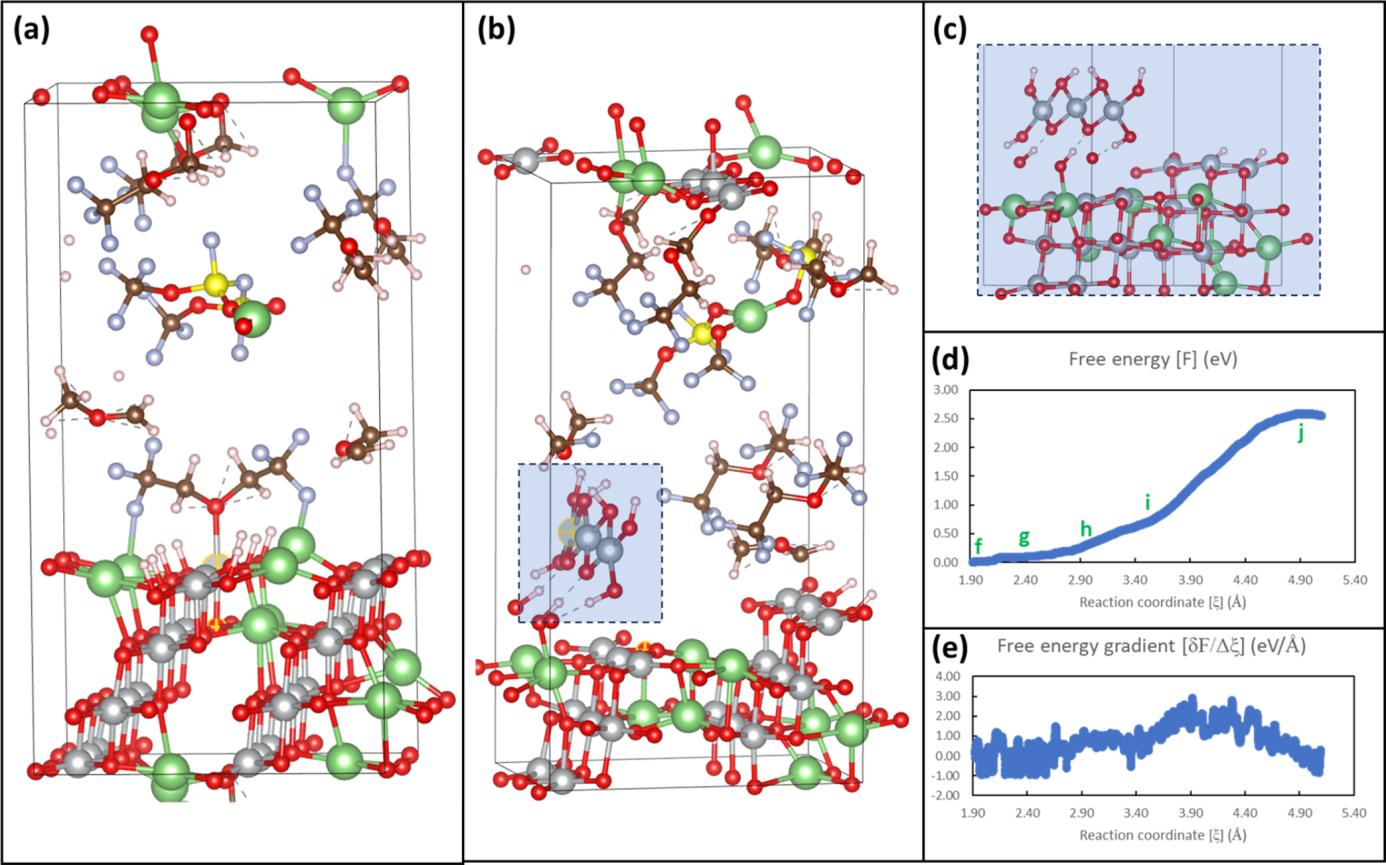
Mechanisms of Ni dissolution from protonated LiNiO_2_ surfaces. (a) Initial configuration (obtained from the AIMD simulation of LHCE decomposition on Li_0.5_NiO_2_ surfaces), where yellow circles highlight the constrained Ni and O_sub_ atoms; (b) final configuration, where the dissolving NiOOH_*x*_ species is above the surface; (c) enlarged view of the dissolving NiOOH_*x*_ species; (d) free-energy profile of surface Ni atoms with the Ni–O_sub_ bond distance as the reaction coordinate (points (f–j) correspond to the panels displayed in Figure 3); (e) averaged free-energy gradient profile. The color code is the same as in Figure 1.

The initial and final configurations of Ni dissolution from protonated Li_0.5_NiO_2_ surfaces are shown in Figure 2a,b. The constrained Ni atom is initially coordinated with four protonated O_s_ atoms and one O_sub_ atom as seen in Figure 2a. The initial Ni–O_sub_ bond length is 1.921 Å. This bond distance increased with a velocity of 0.5 Å/ps to progressively pull the Ni ion off its initial site during the c-AIMD simulations. Figure 2d,e depicts the free energy and averaged free-energy gradient profiles as a function of the Ni–O_sub_ bond length (ξ). The dissolution is considered to be complete when the free energy plateaus at the Ni–O_sub_ bond distance of about 5 Å. It is found that the energy barrier of Ni dissolution from protonated Li_0.5_NiO_2_ surfaces is 2.58 eV. The final configuration of Ni dissolving in the form of a NiOOH_*x*_ fragment is shown in Figure 2b, with the enlarged view of the formation of β-NiOOH^34^ -like species depicted in Figure 2c. The obtained Ni dissolution computational results are consistent with experiments, showing that the NiOOH_*x*_ film was formed at LiNiO_2_ surfaces during the initial electrochemical cycles by the intercalation of H^+^ ions, which triggers the formation of a spinel-like phase (Ni_3_O_4_) at the subsurface.^32^

**Figure 3:**
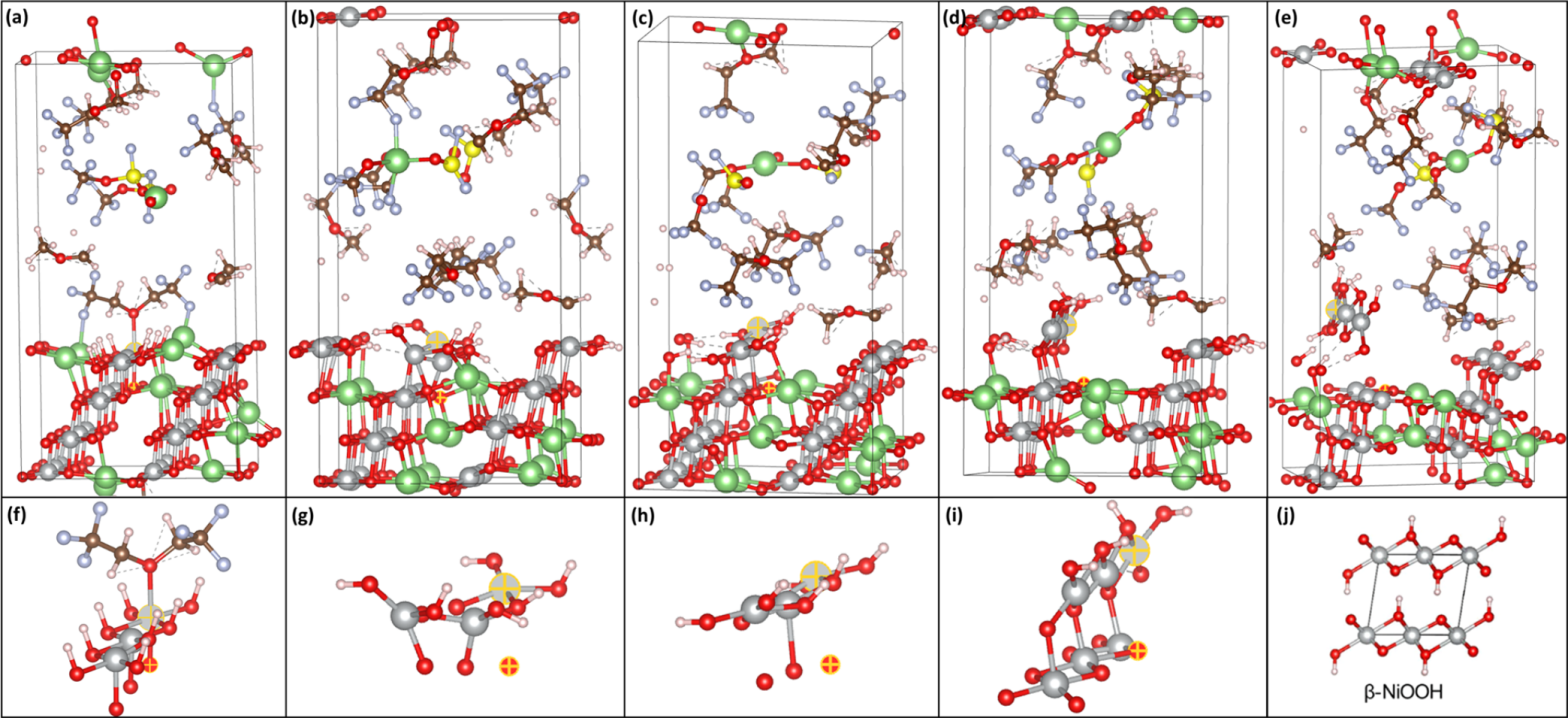
Mechanisms of Ni dissolution from protonated LiNiO_2_ surfaces. (a–e) Representative trajectory of Ni dissolution from the surface-bound state to the dissolved state; (f–i) corresponding bonding structures of the constrained Ni atom (highlighted with yellow circles) upon dissolution; (j) structure of β-NiOOH with a staggered arrangement of intercalated protons.

Figure 3 shows the representative trajectories as the Ni ion separates from the surface with increasing Ni–O_sub_ bond distance, revealing the bond-breaking events during Ni dissolution. The correlation between the free-energy profile shown in Figure 2d and representative events during Ni dissolution from protonated LiNiO_2_ surfaces shown in Figure 3 is summarized in Figure S4. It is seen that Ni dissolution starts with the breakage of the constrained Ni–O_sub_ bond, as depicted in Figure 3g, when ξ increases to 2.42 Å, as seen in Figure 2d, and costs less than 0.1 eV. Further increase of the constrained Ni–O_sub_ bond leads to consecutive breaking of the two neighboring Ni–O_sub_ bonds, as seen in Figure 3h, that builds up the layered NiO_2_ unit (three Ni atoms and six O atoms). Figure 2d shows that the energy barrier for breaking the first neighboring Ni–O_sub_ bond is determined to be 0.37 eV when ξ increases to 3.05 Å, and the breaking of the second neighboring Ni–O_sub_ bond exhibits a barrier of 0.78 eV when ξ increases to 3.60 Å. After all of the Ni–O_sub_ bonds are broken, the two neighboring Ni atoms move together with the constrained Ni atom away from the subsurface in the form of an integrated NiO_2_ unit. The breaking of surface O atoms of subsurface Ni atoms is shown in Figure 3(i), which eventually leads to the detachment of Ni in the form of a NiOOH_*x*_ fragment and the formation of β-NiOOH^34^ -like species as seen in Figure 2b,c.

The obtained Ni dissolution mechanism is very different from the events observed in the computational studies of Mn dissolution.^33,35^ The dissolution of Mn is in the form of singular Mn^2+^-ion complexes with decomposed organic electrolytes through the breakage of Mn–O_s_ and Mn–O_sub_ bonds, while neighboring Mn atoms do not move away from the surfaces. F^–^ anions which can come from PF_6_^–^ decomposition can strongly bind to the surface Mn atom, facilitating the dissolution process. And surface protonation results in a substantial reduction of the dissolution barriers.^35^ On the other hand, the breaking of Ni–O_sub_ and O_s_–Ni_sub_ bonds leads to the dissolution of an integrated NiO_2_ unit from the protonated surfaces in the form of β-NiOOH^34^ -like species, as depicted in Figure 3j. To provide more detailed insight into how surface protonation may affect Ni dissolution, we carried out the simulation of Ni dissolution from pristine Li_0.5_NiO_2_ surfaces. We found that protonation significantly decreases the dissolution barrier, consistent with the investigation of transition-metal dissolution from LiNi_0.5_Mn_1.5_O_4_ surfaces.^35^ As seen in Figure S5, the dissolution barrier is reduced by ∼1.5 eV upon surface protonation. This agrees with the observation that protonation contributes to the reduction of Ni and weakens the strength of Ni–O bonding. To explore the effect of degree of cathode delithiation on Ni removal from the surface, we performed similar c-AIMD simulations using simplified models without the incorporation of electrolyte molecules, as seen in Figure S6. The free-energy profile shown in Figure S6(c) demonstrates that the barrier for Ni removal is reduced by ∼2.0 eV upon surface delithiation. Since surface delithiation also increases the favorability of surface protonation (Figure S2), it can be concluded that Ni removal from the surface is facilitated by cathode delithiation and is further facilitated by the presence of the electrolyte as shown in Figure S4. Figure S6 in the Supporting Information depicts the free-energy profile of Ni removal from Li_1.0_NiO_2_ and Li_0.5_NiO_2_ surfaces.

### 3.3 Proton-Induced Structural Degradation of LiNiO_2_ Surfaces

**Figure 4:**
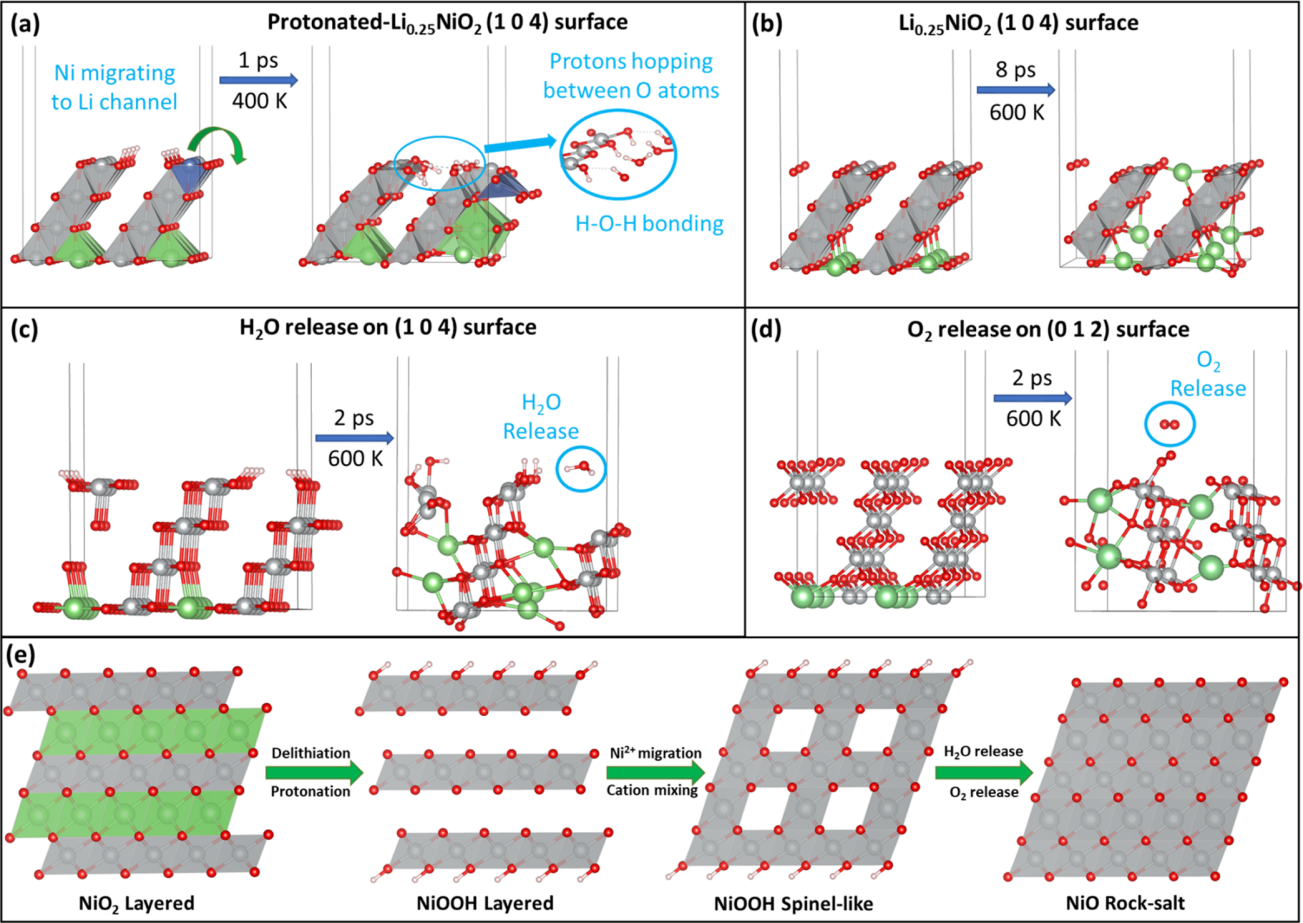
Effect of protonation on the structural changes of LiNiO_2_ surfaces. (a) Ni migrating to a Li vacant site and surface protons hopping between O atoms on Li_0.25_H_0.25_NiO_2_ (104) surfaces after 1 ps of AIMD simulations at *T* = 400 K; (b) structural evolution of proton-free Li_0.25_NiO_2_ (104) surfaces after 8 ps of AIMD simulations at *T* = 600 K; (c) H_2_O release from Li_0.25_H_0.25_NiO_2_ (104) surfaces after 2 ps of AIMD simulations at *T* = 600 K; (d) O_2_ release from Li_0.25_NiO_2_ (012) surfaces after 2 ps of AIMD simulations at *T* = 600 K; (e) structural degradation pathway. The color code is the same as in Figure 1.

Despite a clear correlation between structural degradation and capacity fading of LIB cathodes established in experiments,^36,37^ the microscopic understanding of LiNiO_2_ cathode degradation is still insufficient. To investigate the effect of surface protonation on the structural changes of LiNiO_2_ surfaces, we built a model of protonated (H content = 0.25) delithiated Li_0.25_NiO_2_ (104) surfaces, as shown in Figure 4a. Ni migration (highlighted with blue triangles) from the TM site into a Li vacancy site was identified after 1 ps of AIMD simulations at *T* = 400 K. Such a cation migration phenomenon was also reported during electrochemical cycles, namely, the Ni/Li cation mixing problem.^38−40^ Because of the similar ionic radii of Li^+^ (0.76 Å) and Ni^2+^ (0.69 Å), Ni^2+^ tends to occupy Li sites during cycling, which hindered the Li-ion mobility, thus reducing the rate capability of Ni-rich cathodes.^41,42^ However, there is no such phenomenon found in proton-free surfaces even at the high temperature of *T* = 600 K, as seen in Figure 4b, which suggests that minimizing surface protonation by using a less reactive electrolyte (e.g., LHCE) is an efficient strategy for achieving better stability of Ni-rich cathodes.

Surface protons are found to be mobile as some protons were transferred to O_s_ atoms of neighboring NiO_2_ units and to O_s_ atoms on subsurface layers. The mobility of protons indicates the possibility of intercalation of protons into the lattice and thus leads to the reduction of lattice Ni atoms. The formation of H–O–H bonds was identified by O bonding with a surface Ni atom. When the simulation temperature was elevated to *T* = 600 K, as seen in Figure 4(c), H_2_O was found to release from the surface by breaking the Ni–O bond. Meanwhile, the formation of O–O bonding and the release of O_2_ were observed on Li_0.25_NiO_2_ (012) surfaces after 2 ps of AIMD simulations at *T* = 600 K. The thermal instability of LiNiO_2_ was also identified by the thermogravimetric analysis of LiNiO_2_ after atmospheric exposure accompanied by H_2_O and O_2_ evolution.^43^ The whole process was quantified by the following equation
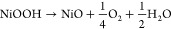
1

The formation of NiO suggests that surface protonation is an important cause of the phase transition of LiNiO_2_ from layered to spinel and eventfully to rock salt. According to a previous study on LiNiO_2_-based electrodes in aqueous electrolyte systems,^32^ spinel-like (Ni_3_O_4_) was more easily formed because of formation of the NiOOH_*x*_ film, as NiOOH is a promising electrocatalyst for the oxygen evolution reaction (OER).^44,45^ Xu and co-workers proposed that the origin of the enhanced OER activity is due to the formation of delithiated LiNiO_2_/NiOOH heterojunction, which involves the formation of a superoxo-like intermediate (NiOO*) that can be stabilized by the Li vacancies in delithiated LiNiO_2_. Deprotonation of NiOOH is charge-compensated by the formation of localized electron holes on O atoms, which act as electrophilic centers that promote the OER.^46^

Because there are many other sources of proton generation than the vigorous decomposition of electrolyte solvent,^47^ protonation can also happen to layered metal oxides in the bulk.^48^ Hongo and co-workers examined the structural changes in the crystal structure of Li_1–*x*_H_*x*_NiO_2_ with Li^+^/H^+^ cations.^43^ As shown in Figure S7, the activation energies of Li^+^ diffusion for Li_0.741_NiO_2_, Li_0.704_H_0.037_NiO_2_, Li_0.481_NiO_2_, and Li_0.444_H_0.037_NiO_2_ were determined to be 0.567, 0.655, 0.193, and 0.266 eV, respectively. The increase in the activation energy was caused by a decrease in the Li layer thickness, which reduced the ionic conductivity. Therefore, we believe that protonation induces rapid degradation of LiNiO_2_ to transform from layered to spinel and eventually to rock salt, as seen in Figure 4e. This transformation is the combined impact of cation disorder and oxygen loss and is accelerated at a high delithiation state and high temperatures. The following equations describe the phase transition
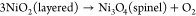
2
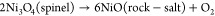
3

### 3.4 Reduction of Dissolved Ni^2+^ on LMA Surfaces

**Figure 5:**
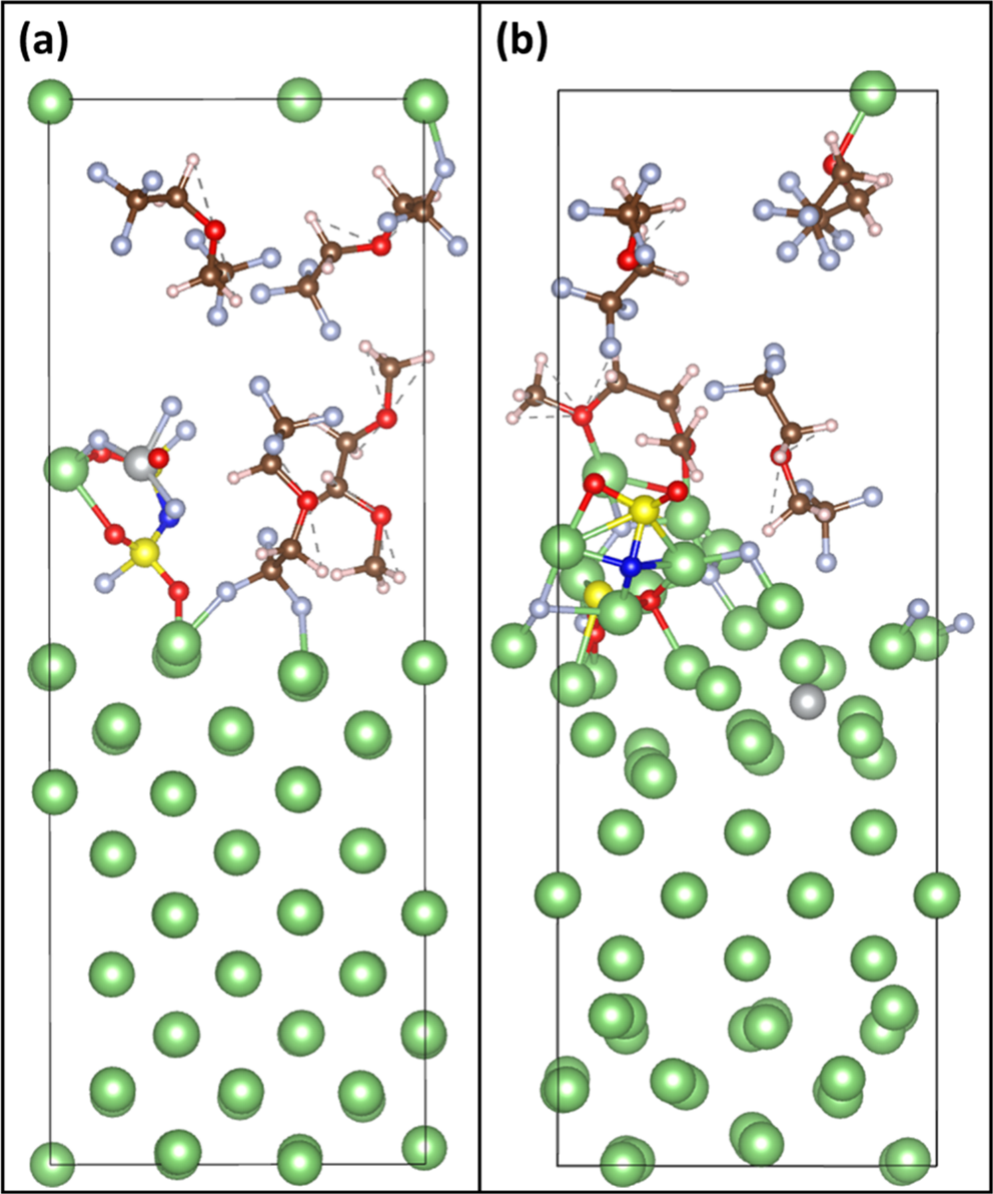
Reduction of dissolved Ni^2+^ on LMA surfaces. (a) Initial configuration of LHCE with NiF_3_^–^ on LMA surfaces; (b) final configuration with Ni reduced to the metallic state after 10 ps of AIMD simulations at *T* = 330 K. The color code is the same as in Figure 1.

Apart from the various degradation mechanisms at the cathode side, detrimental reactions in the anode could also cause further battery performance decay. One of the cross-talk problems is the migration of dissolved TM ions from layered-structured Ni-rich cathodes to the anode side, which could accelerate the failure of lithium-ion batteries.^49^ To investigate the impacts of dissolved Ni^2+^ on the solid-electrolyte interphase (SEI) of lithium-metal anode (LMA) surfaces, we built a model of LHCE with NiF_3_^–^ in contact with the (100) facet of a Li metal slab as seen in Figure 5a. AIMD simulations were employed to examine the evolution of dissolved Ni^2+^ following the study of LHCE decomposition on LMA surfaces.^28^ The dissolved Ni^2+^ ion was added to LHCE in the form of NiF_3_^–^, as NiF_3_^–^ was the fragment identified by time-of-flight secondary ion mass spectrometry (ToF-SIMS) in Li|LiNiO_2_ cells.^50^ NiF_3_^–^ defluorination was found within 2 ps of AIMD simulations, leading to the formation of LiF on LMA surfaces. After the decomposition of LiFSI, the reduction of Ni^2+^ was identified at around 6 ps after AIMD simulations. Ni reduced from the oxidation state of +2 to the metallic state, and the final configuration is shown in Figure 5b, where the reduced Ni atom deposits on LMA surfaces. The reactivity of LHCE was not affected by Ni impurity. LiFSI decomposition is the main contributing component of SEI. DME and BTFE were stable during the 10 ps of AIMD simulations. The reduction of dissolved Ni^2+^ was also reported in experimental studies.^51^ The reduced Ni atoms were found to incorporate into small clusters, causing impeded Li-ion transport and the formation of dead Li, which may contribute to the failure of LIBs. The presence of metallic Ni can lead to changes in the SEI properties. Further related analyses will be discussed in a future work.

## 4 Conclusions

In this work, we conducted a systematic analysis of electrolyte deprotonation-induced structural degradation of the LiNiO_2_ cathode. Simulations of electrolyte decomposition on (104) surfaces demonstrated that proton transfer from electrolytes to cathode surfaces leads to the reduction of Ni cations and weakening of Ni–O bonds. Such proton transfer is related to electrolyte oxidation which depends on the solvation structure and deprotonation reaction energetics. The higher surface reactivity of EC/PC compared to LHCE and the dependence of solvent availability on the solvation structure suggest that optimizing the electrolyte solvation structure as well as electrolyte composition should be strategic factors to achieve better cycling stability for Ni-rich cathodes. The c-AIMD simulations of Ni dissolution revealed that dissolving Ni cations result in the formation of NiOOH*_x_* structures upon surface protonation. It is found that the energy barrier of Ni dissolution from protonated Li_0.5_NiO_2_ surfaces is reduced by ∼1.5 eV upon surface protonation, suggesting that minimizing electrolyte deprotonation by using less reactive LHCE electrolytes is a promising solution to preventing degradation effects in Ni-rich cathodes. The resultant NiOOH_*x*_ formed on the cathode surfaces stimulates Ni migration, which hinders Li^+^-ion mobility, resulting in higher resistance and lower rate capability. Further, NiOOH is a well-known catalyst for the evolution of O_2_ from the cathode. Evolution of H_2_O and O_2_ triggers the phase transition of LiNiO_2_ from layered to rock salt, thus leading to the structural degradation of Ni-rich cathodes. Moreover, migration of the dissolved Ni^2+^ to the Li anode side and reduction into a metallic state on anode surfaces may induce unwanted changes to the anode SEI electron conductance behavior.

## Supporting Information Available

AIMD simulation scheme; energetics of electrolyte deprotonation reaction on Li_1.0_NiO_2_ and Li_0.5_NiO_2_ (104) surfaces; DME deprotonation on Li_1.0_NiO_2_ and Li_0.5_NiO_2_ (104) surfaces; magnetic moments of Ni atoms in the simulations of DME deprotonation on Li_1.0_NiO_2_ and Li_0.5_NiO_2_ (104) surfaces; correlation between the free-energy profile and representative trajectories of Ni dissolution from protonated Li_0.5_NiO_2_ surfaces; free-energy profiles of Ni dissolution from pristine and protonated Li_0.5_NiO_2_ surfaces; free-energy profile of Ni dissolution from Li_1.0_NiO_2_ and Li_0.5_NiO_2_ surfaces; and energy profiles of Li diffusion path of Li_1–*x*_H_*y*_NiO_2_ (PDF)
