# Supplementary material for: Exploring the Mechanisms of LiNiO2 Cathode Degradation by the Electrolyte Interfacial Deprotonation Reaction
Source: ACS Appl Mater Interfaces. 2024 Oct 4;16(41):55258–66. doi: 10.1021/acsami.4c10458 (PMC11492167; doi:10.1021/acsami.4c10458)
Supplement: Supplementary file 1 — am4c10458_si_001.pdf [file am4c10458_si_001.pdf]

Supporting Information for

Exploring the Mechanisms of LiNiO2 Cathode Degradation by the Electrolyte Interfacial Deprotonation Reaction

Yu Zheng[1,2] and Perla B. Balbuena[1,2,3,*]

[1]Department of Chemical Engineering, [2]Department of Chemistry, [3]Department of Materials Science and Engineering, Texas A&M University, College Station, TX, 77843, United States

*e-mail: balbuena@tamu.edu

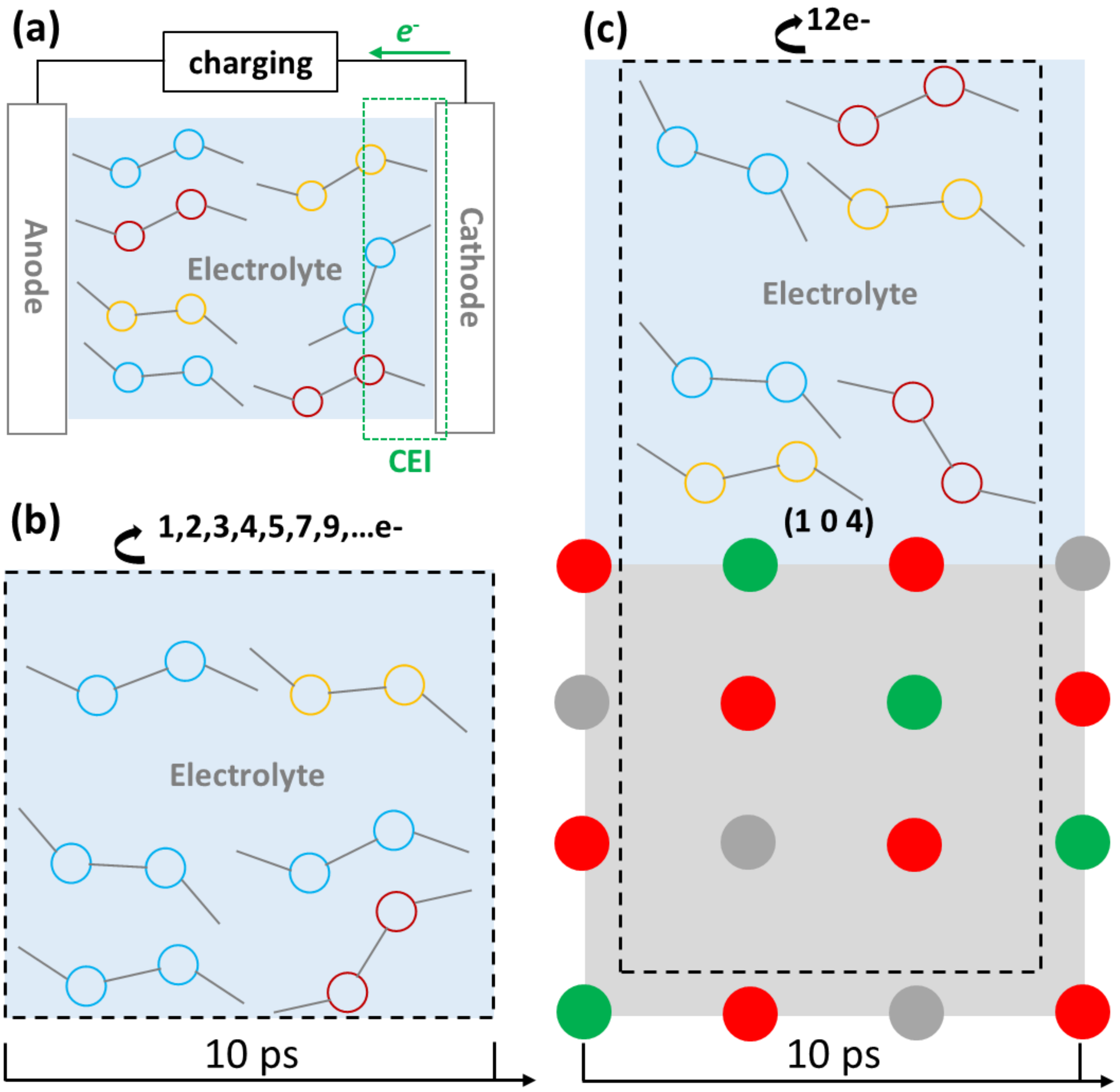

Figure S1. AIMD simulation scheme. (a) Schematic illustration of electrolyte-cathode interfacial region during battery charging. (b) Scheme of creating electron-deficient environments through initially removing different number of electrons (n_{re} = 1, 2, 3, 4, 5, 7, 9, …) from the simulation cell for bulk electrolyte decomposition. (c) Scheme of creating electron-deficient environments through electron removal (n_{re} = 12) from the simulation cell for electrolyte interfacial decomposition on $LiNiO_2$ (1 0 4) surfaces. The color code is the same as in Figure 1.

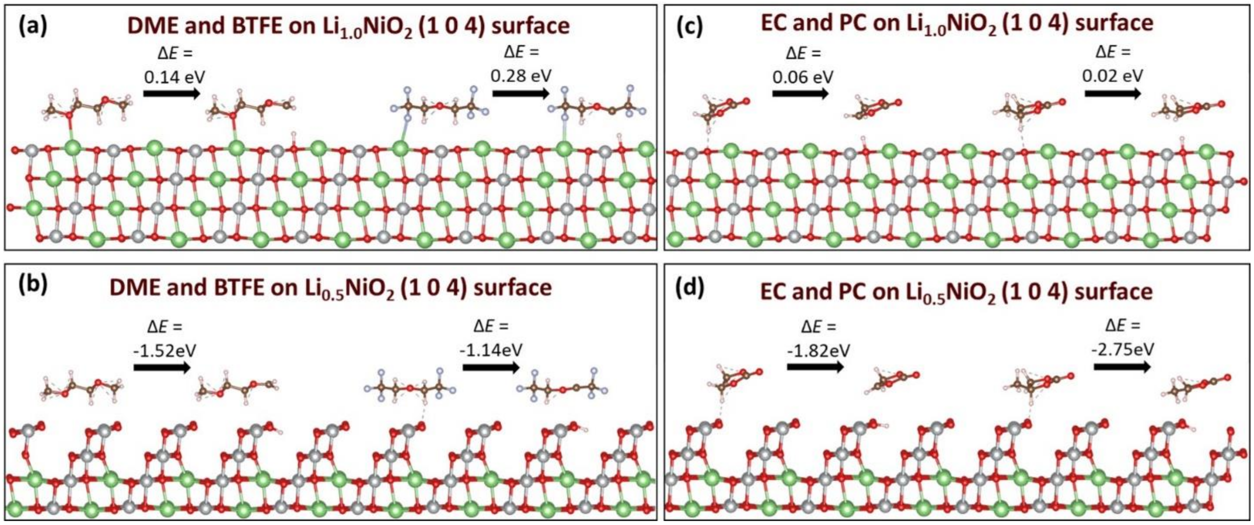

Figure S2. Reaction energies (in eV) of electrolyte deprotonation reaction on (a, c) $Li_{1.0}NiO_2$ and (b, d) $Li_{0.5}NiO_2$ (1 0 4) surfaces. The color code is the same as in Figure 1.

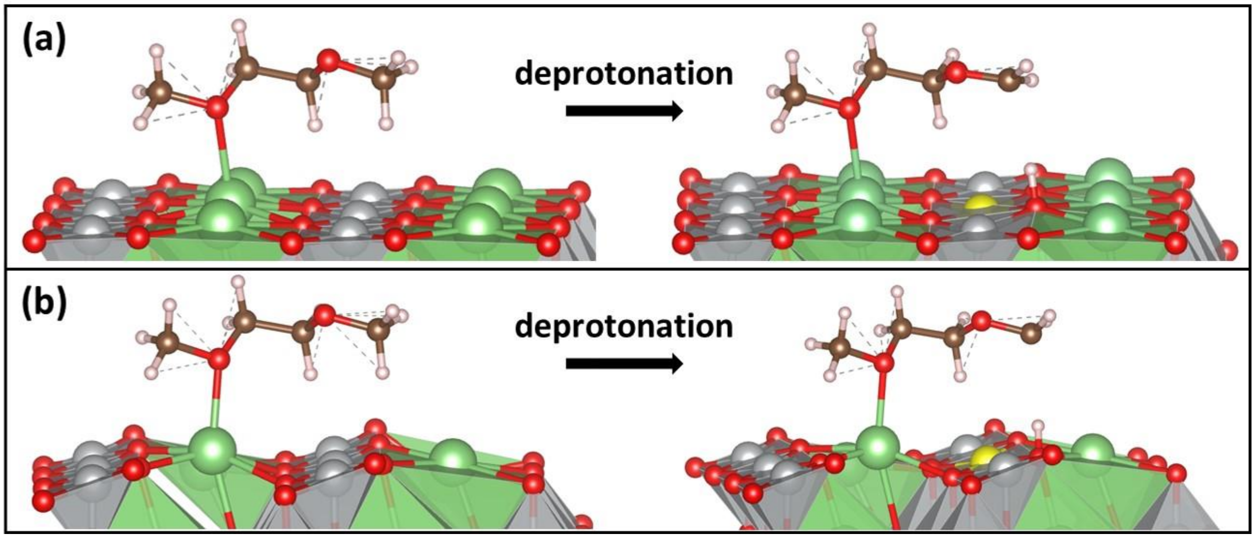

Figure S3. DME deprotonation on (a) $Li_{1.0}NiO_2$ and (b) $Li_{0.5}NiO_2$ (1 0 4) surfaces (reduced Ni atoms are highlighted in yellow and subsurface layers aren't shown). The color code is the same as in Figure 1.

Table S1 Magnetic moments surface Ni atoms (highlighted in yellow in Figure S3) of the simulations of DME deprotonation on $Li_{1.0}NiO_2$ and $Li_{0.5}NiO_2$ (1 0 4) surfaces. (Ni^{2+}, Ni^{3+} and Ni^{4+} have the electronic configurations (|↑↓|) as t_{2g}^6(|↑↓|↑↓|↑↓|) e_g^2(|↑|↑|), t_{2g}^6(|↑↓|↑↓|↑↓|) e_g^1(|↑||) and t_{2g}^6(|↑↓|↑↓|↑↓|) eg^0(|||), respectively, which corresponds to magnetic moments of 2, 1 and 0.)

$Li_{1.0}NiO_2$					$Li_{0.5}NiO_2$				
	Magnetic moments		Charge State			Magnetic moments		Charge State	
	initial	final	initial	final		initial	final	initial	final
Ni1	1.172	1.249	3	3	Ni1	1.19	1.161	3	3
Ni2	1.192	1.161	3	3	Ni2	0.274	0.043	4	4
Ni3	1.184	1.180	3	3	Ni3	0.362	0.342	4	4
Ni4	1.237	1.242	3	3	Ni4	1.16	1.233	3	3
Ni5	1.245	1.245	3	3	Ni5	1.175	1.204	3	3
Ni6	1.171	1.154	3	3	Ni6	0.195	0.136	4	4
Ni7	1.176	1.214	3	3	Ni7	0.284	0.342	4	4
Ni8	1.252	1.733	3	2	Ni8	1.284	1.557	3	2
Ni9	1.229	1.245	3	3	Ni9	1.183	1.181	3	3
Ni10	1.183	1.163	3	3	Ni10	1.119	1.136	3	3
Ni11	1.189	1.170	3	3	Ni11	0.343	0.129	4	4
Ni12	1.352	1.409	3	3	Ni12	1.385	1.245	3	3
Ni13	1.250	1.254	3	3	Ni13	1.185	1.202	3	3
Ni14	1.163	1.192	3	3	Ni14	0.115	0.059	4	4
Ni15	1.173	1.176	3	3	Ni15	0.36	0.308	4	4
Ni16	1.259	1.422	3	3	Ni16	1.276	1.323	3	3
Ni17	1.237	1.251	3	3	Ni17	1.18	1.206	3	3
Ni18	1.219	1.173	3	3	Ni18	1.145	1.111	3	3
Ni19	1.182	1.180	3	3	Ni19	0.413	0.219	4	4
Ni20	1.233	1.265	3	3	Ni20	1.422	1.143	3	3
Ni21	1.254	1.244	3	3	Ni21	1.183	1.268	3	3
Ni22	1.177	1.170	3	3	Ni22	1.09	1.148	3	3
Ni23	1.184	1.202	3	3	Ni23	0.173	0.336	4	4
Ni24	1.252	1.194	3	3	Ni24	1.225	1.124	3	3

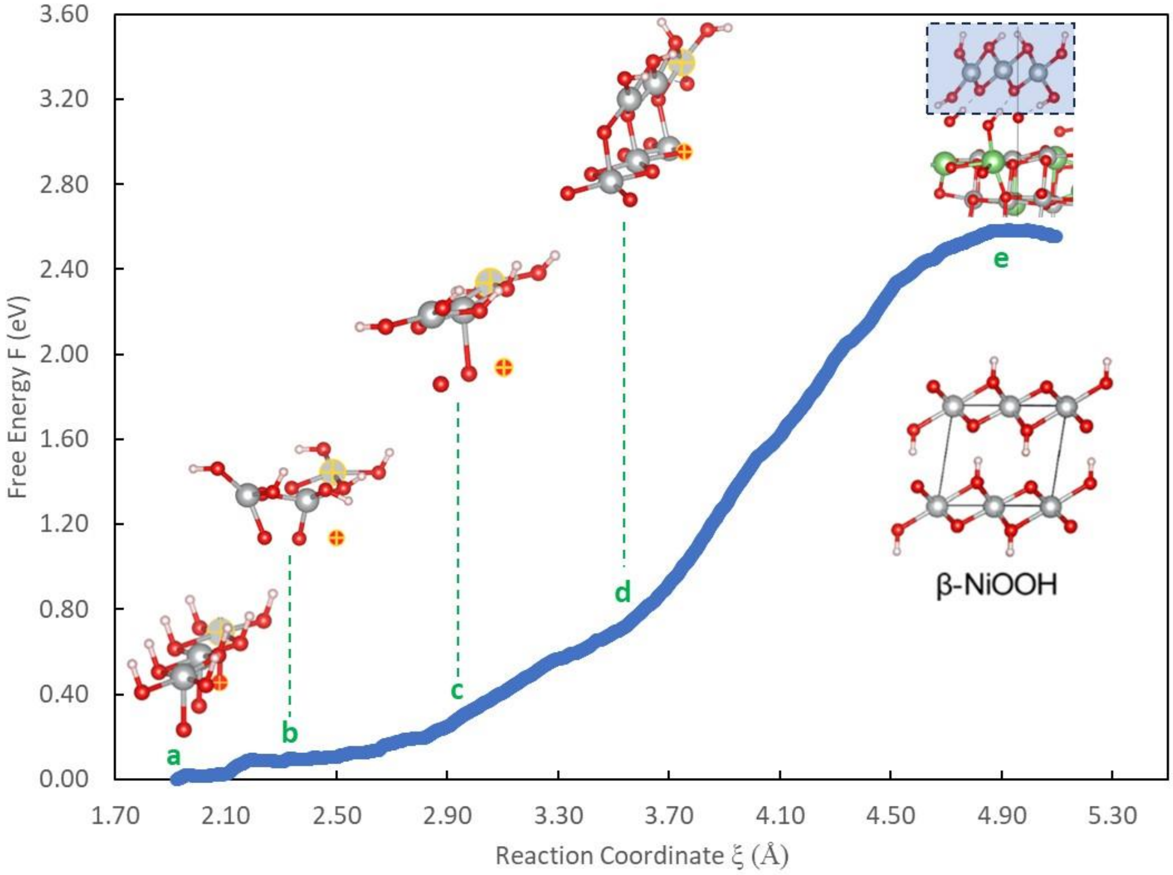

Figure S4. The correlation between the free energy profile shown in Figure 2 (d) and representative events during Ni dissolution from protonated $LiNiO_2$ surfaces shown in Figure 3.

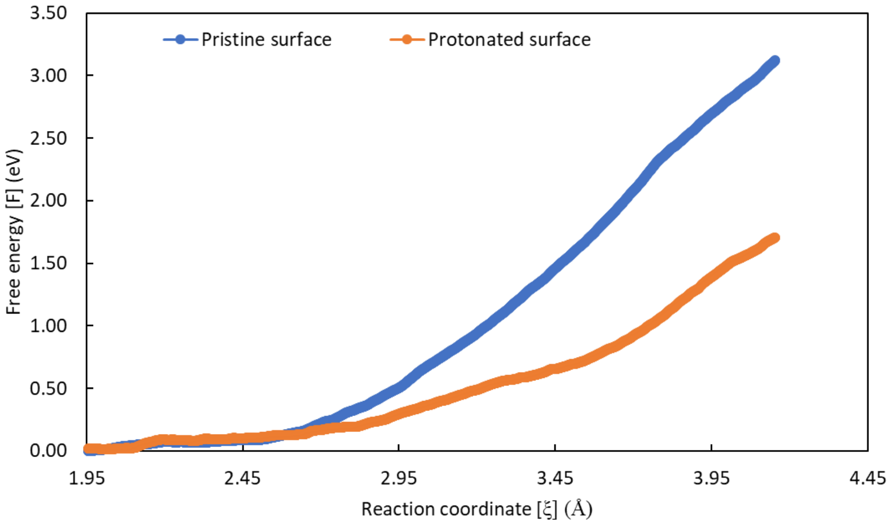

Figure S5. Free energy profiles of Ni removal from pristine and protonated $Li_{0.5}NiO_2$ surfaces.

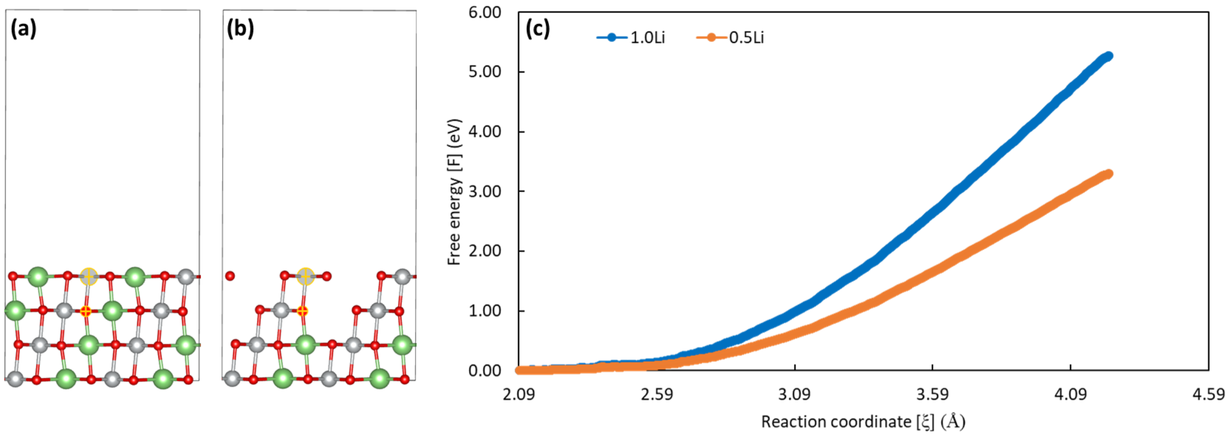

Figure S6. Free energy profile of Ni removal from $Li_{1.0}NiO_2$ and $Li_{0.5}NiO_2$ surfaces using simplified models without the incorporation of electrolytes. (a) simplified model of $Li_{1.0}NiO_2$, (b) simplified model of $Li_{0.5}NiO_2$ surfaces, (c) free energy profile.

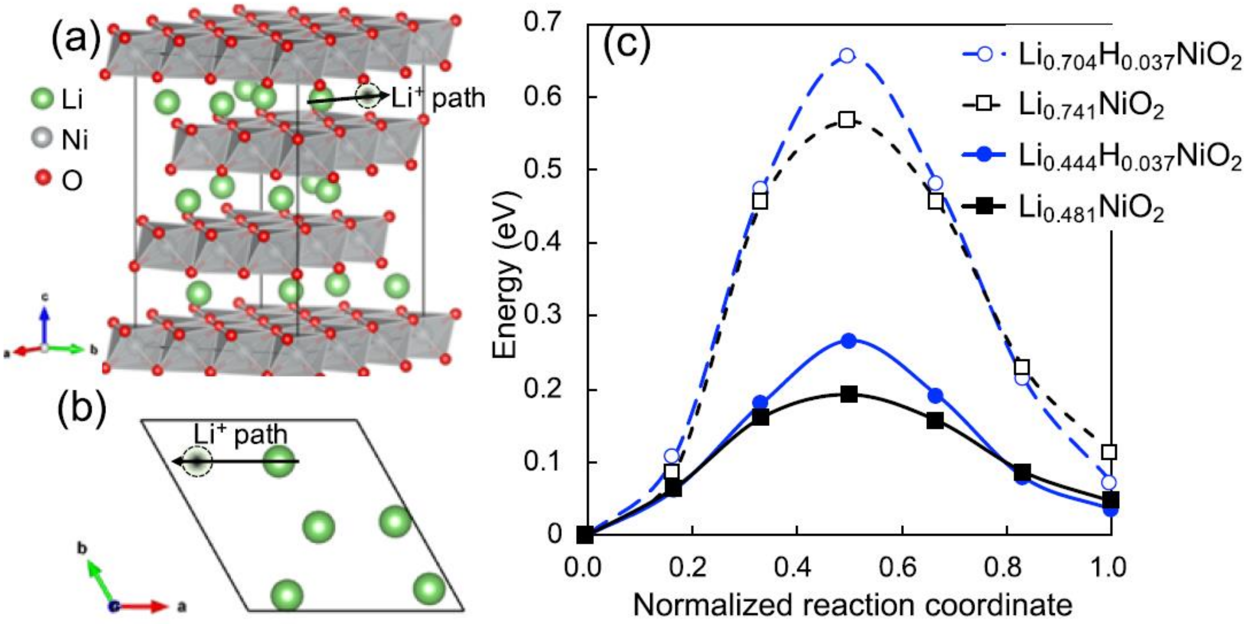

Figure S7. Energy profiles of Li diffusion path of $Li_{1-x}H_yNiO_2$ determined by the climbing image nudged elastic band (CI-NEB) method.[1] (a) Li^+ diffusion path of $Li_{0.481}NiO_2$ by the CI-NEB calculation. (b) Top view of the Li layer, where the Li diffusion path exists in the c-axis direction. (c) Energy profiles of Li diffusion path for $Li_{0.741}NiO_2$, $Li_{0.704}H_{0.037}NiO_2$, $Li_{0.481}NiO_2$, and $Li_{0.444}H_{0.037}NiO_2$. Reproduced from reference [1]. Copyright 2020, American Chemical Society.

References

(1) Toma, T.; Maezono, R.; Hongo, K. Electrochemical Properties and Crystal Structure of Li+/H+ Cation-Exchanged LiNiO2. *ACS Applied Energy Materials* **2020**, *3* (4), 4078-4087. DOI: 10.1021/acsaem.0c00602.
